# Analgesic effects of perioperative acupuncture methods: A narrative review

**DOI:** 10.1097/MD.0000000000035759

**Published:** 2023-10-27

**Authors:** Ling Liu, Guoqing Zhao, Yuchang Dou, Longyun Li, Peng Chen, Tao Li, Ming Gao

**Affiliations:** a Department of Anesthesiology, China-Japan Union Hospital of Jilin University, Changchun, China; b Jilin University, Changchun, China; c Department of Traditional Chinese Medicine, China-Japan Union Hospital of Jilin University, Changchun, China.

**Keywords:** acupuncture, alternative analgesia, alternative medicine, analgesia, complementary medicine, perioperative acupuncture, postoperative pain

## Abstract

Postoperative pain occurs immediately after surgery. The most common perioperative analgesic methods are nerve block, patient-controlled intravenous analgesia, and patient-controlled epidural analgesia. However, overuse of opioid analgesics can cause many adverse reactions including excessive sedation, respiratory inhibition, postoperative nausea, and vomiting. In recent years, many clinical trials have shown that perioperative acupuncture has unique advantages in patients. Perioperative acupuncture can relieve intraoperative pain, improve postoperative pain management, reduce postoperative nausea and vomiting, and shorten the length of hospital stay. This study aimed to confirm the analgesic effect of perioperative acupuncture by reviewing studies on the different methods of perioperative acupuncture and their analgesic effects. The cited literature was searched in English and Chinese from PubMed, China National Knowledge Infrastructure, and Wanfang data, using the following keywords: “perioperative pain,” “acupuncture,” “electroacupuncture,” and “perioperative analgesia.” Studies published from 2005 to 2023 were included. All retrieved papers were read in detail. Perioperative acupuncture has benefits in reducing postoperative pain and opioid need. Although analgesic drugs are still the primary means of postoperative pain control, acupuncture provides a safe analgesic supplement or alternative. This review aimed to assist practitioners in choosing appropriate perioperative acupuncture methods by summarizing the recent literature on the role of different acupuncture approaches for perioperative pain management.

## 1. Introduction

Postoperative pain is a common type of clinical postoperative acute pain, including pain as a result of an operation, visceral trauma, and inflammatory stimulation around nerve endings following traumatic pain. An evaluation of 1490 surgical inpatients in the Netherlands showed that 41% of patients experienced moderate to severe pain on the day of surgery despite the presence of acute pain treatment regimens, and approximately 15% noted moderate to severe pain on the fourth day postoperatively.^[1]^ Local tissue or nerve damage caused by surgery can induce an excessive stress response in the body, activate pain receptors, cause the body to release harmful substances, and cause local or systemic neuro-endocrine changes, thereby causing tachycardia, hypertension, hyperglycemia, immune inhibition, platelet stagnation, and other symptoms. When pain associated with tissue injury and inflammation lasts longer than the expected postoperative healing period (≥2 months), persistent postoperative pain develops, which affects patients’ recovery time and can lead to mental, emotional, and behavioral changes.^[2]^ Currently, commonly used postoperative analgesic methods include intravenous application of opioids by medical personnel, patient-controlled intravenous analgesia (PCIA), patient-controlled epidural analgesia, and nerve blocks. However, opioids may cause adverse events, such as constipation, excessive sedation, respiratory inhibition, postoperative nausea and vomiting, and depression,^[3,4]^ and previously opioid-free patients have a high rate of and severe consequences from opioid-related adverse events.^[5]^ Though opioid analgesia remains to be the major method for postoperative pain management, the result of its use has risks for patients even after discharge.^[6]^ Therefore, a multimodal approach for perioperative pain management has been promoted since it reduces dependence on drugs and improves outcomes. Current research suggests that acupuncture can reduce the pain response and increase pain tolerance as a result of various biologically active chemicals related to peripheral and spinal regions.^[7]^ Although its perioperative analgesia mechanism is not clear, the safety and promising advantages of acupuncture as a complementary and alternative medicine in perioperative analgesia has been confirmed in several clinical trials in recent years. Perioperative acupuncture analgesia is the use of acupuncture to reduce intraoperative pain stimulation and opioid need, improve postoperative pain management, reduce postoperative nausea and vomiting, and shorten hospitalization time. This is achieved by selecting acupoints along meridians, local acupoints, syndrome differentiation, and symptomatic acupoints according to different diseases and surgical sites.^[8]^ This review aimed to describe the postoperative analgesic effects of different perioperative acupuncture methods in recent years and to suggest a multimodal approach for perioperative pain management by using acupuncture for further research.

## 2. Methods

### 2.1. Selection criteria and search strategy

A search was conducted manually in English and Chinese using PubMed, China National Knowledge Infrastructure, and Wanfang data, using the following keywords: “perioperative pain,” “acupuncture,” “electroacupuncture,” and “perioperative analgesia.” Studies published from 2005 to 2023 were included. All retrieved papers were read in detail.

Since this is a narrative review, ethical approval was not required.

## 3. Discussion

In all the typical cases analyzed, 22 studies confirmed that perioperative acupuncture had a significant analgesic effect, fifteen studies stated that it reduced or delayed the need for analgesic drugs, and 5 studies concluded that there was no significant pain reduction in the acupuncture group compared with the control group. Perioperative acupuncture has benefits in reducing postoperative pain and opioid need. Although analgesic drugs are still the primary means of postoperative pain control, acupuncture can provide a safe analgesic supplement or alternative. Due to the lack of multi-center and large-sample trials, the effect of perioperative acupuncture is still unclear.

There are many methods of acupuncture commonly used for pain management, but few cases explored different acupuncture methods for the same type of operation. Therefore, we only classified acupuncture methods in this review to explore the analgesic effect of the different perioperative acupuncture methods.

### 3.1. Traditional filiform needle acupuncture

Traditional filiform needle acupuncture refers to the use of different acupuncture techniques to insert filiform needles into the patient’s skin causing mechanical stimulation of acupoints to produce anesthetic and analgesic effects. Unlike using sterile press needles, the filiform needles are not kept in place for a few days; they are removed after few minutes.

Several clinical studies have shown that acupuncture can reduce the degree of pain during the postoperative acute pain period. A systematic review and meta-analysis involving 682 patients suggested that patients who received acupuncture or related techniques had less postoperative pain 1 day postoperatively, and their opioid analgesic consumption was less compared to the control group (*P* < .001).^[9]^ However, subgroup analysis indicated that, although the degree of postoperative pain in the acupuncture group was lower than that in the control group, the 2 groups were similar in reducing opioid analgesic use (*P* ≥ .412).^[9]^

In a retrospective study on postoperative analgesia after lumbar degenerative disc surgery, 96 patients divided into 3 groups received either acupuncture, patient-controlled analgesia (PCA), or routine analgesics for pain control. The results demonstrated that from the first postoperative day to the second, the visual analogue scale (VAS) pain scores of all 3 groups decreased significantly (all, *P* < .05), and there was no significant difference between them. However, the fourth day VAS score of the PCA group was significantly higher (*P* = .026), and the sixth day VAS score of the acupuncture group was significantly lower (*P* = .047) than that of other groups, and there was no significant difference in the decrease of VAS scores among the 3 groups over 6 postoperative days.^[10]^ This suggests that acupuncture might be as effective as traditional analgesia and PCA for adjuvant pain management postoperatively for lumbar degenerative disc disease. Interestingly, when the surgical results were assessed on the day of discharge, 26 patients (68.4%) in the acupuncture group reported either complete pain relief or significant improvement after operation, compared to 51.9% in the PCA group and 37.5% in the routine analgesia group (*P* = .024). These results demonstrated that patients who received acupuncture treatment reported either complete resolution or significant improvement of their back pain after the lumbar degenerative disc surgery in comparison to the routine analgesia and PCA groups.^[10]^ In another randomized controlled trial in 2023, a group of 58 patients undergoing lumbar spinal fusion were randomized to receive acupuncture before and after operation versus routine anesthesia alone. Though acupuncture was not shown to significantly improve pain scores (*P* > .05), the dosage of sufentanil and remedial analgesia within 48 hours after operation were significantly lower in the group receiving acupuncture (*P* < .01).^[11]^

Two randomized trials showed that the use of traditional filiform needle acupuncture decreased pain in children with pain post-tonsillectomy. In a randomized, controlled, single-blinded study, a group of 60 children aged 3 to 12 were randomized to receive conventional postoperative analgesia and the same regime plus postoperative acupuncture, and the Wong-Baker Faces visual analogue pain scale was utilized to assess pain. In the group that received acupuncture, pain decreased significantly after the second and third treatments postoperatively (*P* ≤ .01), and the requirement of analgesic drugs also reduced in comparison to the control group (*P* = .02).^[12]^ In another prospective randomized clinical trial, 251 children aged 2 to 10 were randomly assigned to either control or acupuncture groups. Children in the acupuncture group had less postoperative pain when they received acupuncture after anesthesia induction. The pain scores from 2 to 12 hours postoperatively in the acupuncture group were significantly lower than those in the control group (*P* = .001–.044), but no difference was found between 18 and 24 hours postoperatively or after discharge at home (*P* > .05). Though no difference was noted in the oxycodone requirements between the 2 groups in the hospital, a statistical reduction was showed on day 2 at home (*P* = .035).^[13]^

### 3.2. Transcutaneous electrical acupoint stimulation

Transcutaneous electrical acupoint stimulation (TEAS) is different from electroacupuncture and traditional acupuncture. Instead of placing filiform needles, surface electrodes are used to stimulate acupoints with an electric current.

A multicenter randomized clinical trial involving 568 patients undergoing mastectomy investigated the effect of percutaneous acupoint stimulation before general anesthesia induction on post mastectomy pain. The results showed that the consumption of remifentanil during the operation and the amount of nausea and vomiting in the 24 hours after operation in the combined acupoint group were lower than those in the sham group (*P* < .001). Also, the post mastectomy pain at 6 months after surgery was decreased in the combined acupoint group (*P* = .001).^[14]^

Furthermore, TEAS has a promising role in acute postoperative pain management in patients undergoing abdominal pain after surgical operation. A meta-analysis of 28 randomized controlled trials involving 2787 participants showed that TEAS could effectively relieve short-term pain after laparoscopic surgery, reduce the dosage of rescue analgesics after surgery, improve the quality of life of patients, and shorten the length of hospital stay (all, *P* < .001). There were no serious adverse events related to TEAS. Therefore, the clinical application of TEAS is safe and effective.^[15]^ Another randomized controlled trial showed that patients using TEAS before anesthesia induction had a significantly lower incidence of abdominal pain after colonoscopy (*P* = .007), and the incidence of abdominal distension was lower in the TEAS group (*P* = .032) than in the control group.^[16]^ Moreover, Szmit et al conducted a randomized, placebo-controlled study to assess analgesia requirement with TEAS in 71 patients after inguinal hernia repair. Patients who received TEAS showed significantly lower VAS pain scores (*P* < .001) and less total analgesia consumption in the postoperative period (*P* < .001) than others.^[17]^ Another prospective, randomized, placebo-controlled trial that included 60 patients undergoing gynaecological laparoscopic surgery demonstrated that patients who received TEAS for 30 minutes at the acupoints of Baihui (GV20), Yingtang (EX-HN-3), Zusanli (ST36), and Neiguan (PC6) before anesthesia had lower VAS scores on postoperative days 1 and 2 (*P* < .05).^[18]^

TEAS is also a beneficial noninvasive pain management method for thoracic surgery. Jiheng Che et al conducted a randomized, double-blind, controlled experimental study on thoracoscopic pulmonary resection. The TEAS group received different degrees of TEAS before anesthesia induction, during the surgical procedure, and after the surgery. The sham-TEAS group also received sham stimulation before and after surgery induction as mentioned. The average VAS score at 6, 24 and 48 hours after surgery in the TEAS group was significantly lower than that in the sham-TEAS group (*P* < .01). The consumption of sufentanil at 6, 24 and 48 hours during PCIA in the TEAS group was significantly decreased compared with the sham-TEAS group (all, *P* < .001). Therefore, TEAS could offer a feasible and effective approach in the reduction of analgesic and pain management in patients undergoing thoracoscopic pulmonary resection.^[19]^ Moreover, Shun Huang et al conducted a randomized, double-blinded, placebo-controlled trial to explore the effect of TEAS used in different frequencies on video-assisted thoracic surgical patients. Eighty patients were randomly divided into 4 groups: control, 2/100, 2, and 100 Hz. The 3 experimental groups received TEAS at 2 Hz, alternating frequency between 2 and 100 Hz or 100 Hz frequencies for 30 minutes before induction, during the surgery and after surgery. The 2/100 group had the lowest opioid consumption (control, *P* ≤ .001; 2 Hz, *P* ≤ .001; 100 Hz, *P* = .026) and lower VAS score than the control group (*P* = .047).^[20]^

### 3.3. Electroacupuncture

Electroacupuncture (EA) stimulates needles with an electrical current using an electroacupuncture machine after the needles are placed to “Deqi” to prevent or treat diseases.^[21]^ To date, multiple studies have shown that EA can reduce the postoperative pain response in perioperative patients.

A systematic review and meta-analysis by Park et al^[22]^ covering 11 cases of thoracotomy (including resection of esophageal cancer, elective lobectomy, and heart valve replacement) showed that the pain score 24 hours postoperatively and the total dose of analgesics taken in the EA group were significantly lower than those of the 2 control groups. The pain score 24 hours postoperatively of EA group showed a standard mean difference of −0.98 (95% confidence interval [CI]: −1.62 to −0.35) and −0.94 (95% CI: −1.33 to −0.55) compared to the sham and conventional analgesia groups, respectively. The standard mean difference values of analgesic consumption of the EA and sham groups were −0.95 (95% CI: −1.42 to −0.47) and −1.96 (95% CI: −2.82 to −1.10), respectively, compared to conventional analgesia.^[22]^ In contrast, a subgroup study by Yu et al^[23]^ on cardiac surgery showed no significant difference in static and dynamic VAS scores between the electroacupuncture combined medicine group and the conventional analgesia group (*P* > .05). The injection dose of dexmedetomidine and morphine hydrochloride significantly decreased in the electroacupuncture combined medicine group (*P* < .05).

In addition, EA applied in the perioperative period can improve postoperative pain and the quality of life of patients undergoing lumbar surgery. Liu et al enrolled 62 patients who underwent modified transforaminal lumbar interbody fusion for lumbar spinal stenosis. The study compared those who were administered non-steroidal anti-inflammatory drugs (control group) and those who were administered analgesics combined with EA (experimental group) postoperatively. The results indicated that there was no significant difference in VAS scores between the 2 groups before and 24 hours postoperatively (*P* > .05), but the VAS score of the experimental group was lower than that of the control group at 48 hours and 1 week postoperatively (*P* < .05). This demonstrates that EA may relieve postoperative pain in patients.^[24]^ Another prospective, randomized, parallel controlled study that enrolled 59 patients undergoing lumbar fusion surgery also demonstrated that patients treated with EA postoperatively had lower VAS scores (*P* < .05) and an increased degree of straight leg elevation (*P* < .05) compared to the patients treated with celecoxib.^[25]^

Some trials have also showed the promising role of EA for analgesia of hemorrhoids surgery. A meta-analysis of 5 RCTs comparing EA to a control group demonstrated lower postoperative pain scores in the EA group compared to the control group at 6 (*P* < .001), 12 (*P* < .001), 24 (*P* = .547), and 72 hours (*P* < .001) after surgery.^[26]^ In a prospective randomized clinical trial, 124 patients receiving a procedure for prolapse and hemorrhoids were randomized into EA and control groups. Patients in the EA group received EA at Baliao point (BL31–BL34) and the surgery while patients in the control group receive the surgery only. The results showed that the EA group had lower VAS scores at 8, 24, 48, and 72 hours postoperatively (*P* < .01, *P* < .01, *P* < .01, and *P* < .05, respectively) and lower frequencies of postoperative analgesic drug requirement per patient (*P* < .01) compared with the control group.^[27]^ Another RCT that enrolled 72 patients who had undergone hemorrhoidectomy and randomly received EA or sham acupuncture as a control also demonstrated that EA alleviated post-hemorrhoidectomy pain. The VAS scores of the EA group were significantly lower at 6 and 24 hours after operation and during defecation (*P* < .05) compared to the control group. Furthermore, the EA group also had lower Wong-Baker FACES Pain Rating scores at 5, 7, and 8 hours after treatment and during defecation (*P* < .05) compared with those of the control group.^[28]^

### 3.4. Sterile press needles acupuncture

Sterile press needles acupuncture, unlike traditional filiform needle acupuncture, utilizes sterile press needles inserted shallowly into the skin at acupoints and left for a few days to pass the meridians and promote defensive qi. Retention of needles for a long time plays a role in nourishing Yang; promoting blood circulation; replenishing qi; dredging meridians and collaterals; relieving pain; and treating symptoms and root causes. When the needle is inserted into the skin, there is almost no pain. With the prolongation of the resident imbedding period of the sterile press needle and the pressure generated by intermittent pressing on the acupuncture site, the sterile press needle embedded under the skin produces lasting and stable stimulation to the body. Moreover, it gradually produces soreness and a swelling sensation, similar to the feeling of traditional filiform needle acupuncture and plays a therapeutic role.^[29]^ Retaining the needle for a longer period increases the total amount of acupoint stimulation, prolongs the duration of acupuncture action, and achieves the effect of continuous treatment. This mild and lasting stimulation can enhance the therapeutic effect of acupuncture analgesia and prevent the recurrence of pain.^[30]^ There are different models of sterile press needles, which are selected by the operator according to the acupoints. Acupuncture using this kind of needle and retaining it for a short period, named such as imbedding needling, auricular acupuncture and intradermal needling, are classified in this category.

A randomized controlled trial of performing sterile press needle acupuncture perioperatively with minimally invasive breast mass resections in 60 patients was conducted. The patients were divided randomly into the control group and imbedding needle group (ING). Approximately 30 minutes prior to surgery and after application of local skin disinfectant, the ING had their Neiguan (PC6), Waiguan (SJ5), and Hegu (LI4) acupoints needled. The needles were retained for 24 hours postoperatively, and the patients were instructed to press the acupuncture site intermittently to provide stimulation. The results demonstrated that the VAS scores in the ING were significantly reduced at 1, 2, 4, 6, and 8 hours postoperatively compared to that in the control group, and the number of patients receiving remedial treatment at 24 hours postoperatively was less in comparison to the control group (*P* < .05). Overall, this study concluded that perioperative acupuncture using imbedded needles relieved early postoperative pain, improved emotional state and comfort, and promoted patient recovery.^[31]^

Two randomized trials exploring the use of sterile press needles for cesarean delivery demonstrated relief of pain. One randomized clinical study by Zhang et al investigated the effects of acupuncture in 135 puerpera after cesarean section. The women were randomly divided into 3 groups: the medicated, sham acupuncture, and acupuncture groups. The acupuncture group underwent PCIA with sterile press needle acupuncture and were found to have lesser dynamic incision pain and uterine contraction pain after cesarean section than that of the control and sham acupuncture groups combined (*P* < .05). In the acupuncture group, the dynamic incision pain scores at 8, 12, and 24 hours postoperatively decreased (*P* < .05), and the VAS pain scores of uterine contractions decreased at 8, 12, 24, and 48 hours postoperatively (*P* < .05). Additionally, the lactation score of the acupuncture group increased over 24 to 48 hours postoperatively (*P* < .05).^[32]^ In another randomized controlled trial, 180 female patients scheduled for elective cesarean delivery were randomized into an acupuncture group (n = 60),a placebo group (n = 60), and a nonrandomised standard care group (n = 60). Besides standard pain treatment, patients in the acupuncture and placebo groups received acupuncture with indwelling intradermal needles and placebo needles, respectively. The acupuncture group showed lower mean pain intensity on movement assessed with VRS-11 compared to the placebo group (*P* = .001) and the standard care group (*P* < .001). Moreover, 98% of patients in the acupuncture group were fully mobilized compared with 83% from the placebo group (*P* = .01) and 58% from the standard care group (*P* < .001) on the first day postoperatively.^[33]^

A randomized controlled study including 54 cases of total hip arthroplasty found that sterile press needles could be used to reduce the need for postoperative analgesia. Based on the results of random grouping, patients were allocated to receive auricular acupuncture with sterile press needles (auricular acupuncture [AA] group) or sham acupuncture (control group) in the evening before surgery, and needles were retained for 3 days postoperatively. The time taken for patients to request the analgesic piritramide postoperatively in the AA group was longer than the control group (*P* = .04), and the AA group required 32% less piritramide during the first 36 hours postoperatively (*P* = .004). However, the pain intensity on the VAS-100 scores and the incidence of analgesia-related side effects in the 2 groups were similar.^[34]^ Another randomized controlled trial enrolled 60 patients after total knee arthroplasty who randomly received true acupuncture or sham acupuncture, and the AA needles were retained in situ for 3 days. The results showed that the acupuncture group had less requirement of fentanyl (*P* = .002) compared with the sham group.^[35]^

However, the advantage of sterile press needles acupuncture seems to be based on the type of surgery. A group of 40 patients undergoing open radical gastrectomy were enrolled into a randomized controlled pilot trial. Though both the sham group and acupuncture group (received AA 24 hours before surgery and remained for 6 days) had a declined tendency of postoperative movement-evoked pain scores, there was no statistical difference between the 2 groups at any observed point (*P* = .234–.888). Also, there was no statistical difference of pain scores at rest between the 2 groups within 48 hours of surgery (*P* = .134–.520); however, from the third day, the group that received AA showed significant pain relief compared to the sham group (*P* = .039–.047). Furthermore, no statistical difference was observed for total opioid consumption between 2 groups (*P* = .101).^[36]^ Additionally, this type of acupuncture did not demonstrate pain relief in a randomized controlled trial of thoracotomy. The acupuncture group even showed higher pain scores at day 30 after surgery (*P* = .9). No statistical differences were observed between the acupuncture group and the control group on pain assessments at 60 and 90 days after surgery (*P* = .17 and *P* = .8, respectively).^[37]^

### 3.5. Combined acupuncture

Some studies have applied two or more acupuncture methods simultaneously according to the patient’s specific circumstances, and in some of these studies, this combination was beneficial and easy to apply.

In a single-blind randomized controlled trial, 60 patients aged 3 to 12 years undergoing adenotonsillectomy were randomly treated with either general anesthesia or general anesthesia plus acupuncture. The acupuncture group received both filiform needle acupuncture and AA, which used sterile press needles. The filiform needles were inserted into the skin of the hands and feet <1 cm deep and were rotated counterclockwise to stimulate the acupoints every 5 minutes until the end of the operation, while the sterile press needles were placed on the ear lobe. Both needles were placed after general anesthesia and removed before the patient awakened. The Wong-Baker FACES pain scale was utilized to evaluate their postoperative pain in the post-anesthesia care ward. In the acupuncture group, both the intraoperative haemodynamic status (*P* ≤ .05) and pain control postoperatively significantly improved (*P* = .0001), and the time before the patients requested the first postoperative analgesia was delayed (*P* = .0001).^[38]^

However, the benefits of combined acupuncture are not the same. In a randomized controlled trial from 2016 to 2018, 72 patients who underwent laparotomy for gynaecological diseases were randomly allocated to the acupuncture group, which received both electroacupuncture and auricular acupuncture, or the control group, which received noninvasive sham acupuncture. The degree of postoperative pain was evaluated with the numerical rating scale 0 to 5 days postoperatively. The results indicated that although the acupuncture group had a smaller pain score at rest by 22 hours postoperatively than the control group (*P* = .029), there was no significant difference in the area under the resting pain curve between the 2 groups (calculated from arrival in the ward to the fifth postoperative day) (*P* = .439). Moreover, in all other secondary outcomes, including analgesic consumption, recovery time variables, quality of life, and incidence of opioid-related side effects, there was no significant difference between the 2 groups.^[39]^

## 4. Conclusions

Acupuncture is the treasure of Chinese traditional medicine. For thousands of years, acupuncture has been popularly used in China and gradually spread to the world. Since the Department of Otorhinolaryngology of Shanghai First People’s Hospital used traditional acupuncture at the bilateral Hegu (LI4) acupoint to complete the first reported surgery to relieve pain, acupuncture anesthesia began to flourish. However, with the emergence of new anesthetic drugs and the improvement of surgical technical requirements, acupuncture anesthesia cannot meet the needs for muscle relaxation, complete analgesia, and inhibition of reflexes. Therefore, Professor Xiong Lize of Xijing Hospital put forward the new concept of “acupuncture and drug balance anesthesia,” which refers to the application of combined acupuncture and anesthesia to make patients more stable in the perioperative period and improve their postoperative discomfort.^[40]^

In addition, there is limited literature to compare the interaction of different acupuncture techniques in postoperative pain management. This study reviewed the latest research progress on the role of different perioperative acupuncture methods in reducing postoperative pain and aimed to provide novel solutions for personalized multi-mode analgesia to improve the comfort of perioperative patients. Continued research of perioperative acupuncture with a more robust patient population may further clarify the role of acupuncture in postoperative analgesia, expand its clinical application, take maximum advantage of its effect, and provide novel solutions for postoperative analgesia.

## Acknowledgements

Thanks to the countless pioneers and scientists who passed down the traditional Chinese medicine for making it possible to combine traditional Chinese medicine with modern medicine.

## Author contributions

**Conceptualization:** Yuchang Dou, Ming Gao.

**Funding acquisition:** Guoqing Zhao.

**Investigation:** Ling Liu.

**Methodology:** Ming Gao.

**Software:** Longyun Li.

**Supervision:** Peng Chen.

**Validation:** Tao Li.

**Writing – original draft:** Ling Liu.

**Writing – review & editing:** Longyun Li.
